# Adaptive neighborhood rough set model for hybrid data processing: a case study on Parkinson’s disease behavioral analysis

**DOI:** 10.1038/s41598-024-57547-4

**Published:** 2024-04-01

**Authors:** Imran Raza, Muhammad Hasan Jamal, Rizwan Qureshi, Abdul Karim Shahid, Angel Olider Rojas Vistorte, Md Abdus Samad, Imran Ashraf

**Affiliations:** 1 Department of Computer Science, COMSATS University Islamabad, Lahore Campus, Lahore, 54000 Pakistan; 2 Universidad Europea del Atlántico, Isabel Torres 21, 39011 Santander, Spain; 3 Universidad Internacional Iberoamericana Campeche, 24560 Campeche, Mexico; 4 Universidade Internacional do Cuanza, Cuito, Bié Angola; 5 Department of Information and Communication Engineering, Yeungnam University, Gyeongsan-si, Gyeongsangbuk-do 38541 South Korea

**Keywords:** Computational biology and bioinformatics, Machine learning

## Abstract

Extracting knowledge from hybrid data, comprising both categorical and numerical data, poses significant challenges due to the inherent difficulty in preserving information and practical meanings during the conversion process. To address this challenge, hybrid data processing methods, combining complementary rough sets, have emerged as a promising approach for handling uncertainty. However, selecting an appropriate model and effectively utilizing it in data mining requires a thorough qualitative and quantitative comparison of existing hybrid data processing models. This research aims to contribute to the analysis of hybrid data processing models based on neighborhood rough sets by investigating the inherent relationships among these models. We propose a generic neighborhood rough set-based hybrid model specifically designed for processing hybrid data, thereby enhancing the efficacy of the data mining process without resorting to discretization and avoiding information loss or practical meaning degradation in datasets. The proposed scheme dynamically adapts the threshold value for the neighborhood approximation space according to the characteristics of the given datasets, ensuring optimal performance without sacrificing accuracy. To evaluate the effectiveness of the proposed scheme, we develop a testbed tailored for Parkinson’s patients, a domain where hybrid data processing is particularly relevant. The experimental results demonstrate that the proposed scheme consistently outperforms existing schemes in adaptively handling both numerical and categorical data, achieving an impressive accuracy of 95% on the Parkinson’s dataset. Overall, this research contributes to advancing hybrid data processing techniques by providing a robust and adaptive solution that addresses the challenges associated with handling hybrid data, particularly in the context of Parkinson’s disease analysis.

## Introduction

The advancement of technology has facilitated the accumulation of vast amounts of data from various sources such as databases, web repositories, and files, necessitating robust tools for analysis and decision-making^1,2^. Data mining, employing techniques such as support vector machine (SVM), decision trees, neural networks, clustering, fuzzy logic, and genetic algorithms, plays a pivotal role in extracting information and uncovering hidden patterns within the data^3,4^. However, the complexity of the data landscape, characterized by high dimensionality, heterogeneity, and non-traditional structures, renders the data mining process inherently challenging^5,6^. To tackle these challenges effectively, a combination of complementary and cooperative intelligent techniques, including SVM, fuzzy logic, probabilistic reasoning, genetic algorithms, and neural networks, has been advocated^7,8^.

Hybrid intelligent systems, amalgamating various intelligent techniques, have emerged as a promising approach to enhance the efficacy of data mining. Adaptive neuro-fuzzy inference systems (ANFIS) have laid the groundwork for intelligent systems in data mining techniques, providing a foundation for exploring complex data relationships^7,8^. Moreover, the theory of rough sets has found practical application in tasks such as attribute selection, data reduction, decision rule generation, and pattern extraction, contributing to the development of intelligent systems for knowledge discovery^7,8^. Extracting meaningful knowledge from hybrid data, which encompasses both categorical and numerical data, presents a significant challenge. Two predominant strategies have emerged to address this challenge^9,10^. The first strategy involves employing numerical data processing techniques such as Principal Component Analysis (PCA)^11,12^, Neural Networks^13–16^, and SVM^17^. However, this approach necessitates converting categorical data into numerical equivalents, leading to a loss of contextual meaning^18,19^. The second strategy leverages rough set theory alongside methods tailored for categorical data. Nonetheless, applying rough set theory to numerical data requires a discretization process, resulting in information loss^20,21^. Numerous hybrid data processing methods have been proposed, combining rough sets and fuzzy sets to handle uncertainty^22–41^. However, selecting an appropriate rough set model for a given dataset necessitates exploring the inherent relationships among existing models, presenting a challenge for users. The selection and utilization of an appropriate model in data mining thus demand qualitative and quantitative comparisons of existing hybrid data processing models.

This research endeavors to present a comprehensive analysis of hybrid data processing models, with a specific focus on those rooted in neighborhood rough sets (NRS). By investigating the inherent interconnections among these models, this study aims to elucidate their complex dynamics. To address the challenges posed by hybrid data, a novel hybrid model founded on NRS is introduced. This model enhances the efficiency of the data mining process without discretization mitigating information loss and ambiguity in data interpretation. Notably, the adaptability of the proposed model, particularly in adjusting the threshold value governing the neighborhood approximation space, ensures optimal performance aligned with dataset characteristics while maintaining high accuracy. A dedicated testbed tailored for Parkinson’s patients is developed to evaluate the real-world effectiveness of the proposed approach. Furthermore, a rigorous evaluation of the proposed model is conducted, encompassing both accuracy and overall effectiveness. Encouragingly, the results demonstrate that the proposed scheme surpasses alternative approaches, adeptly managing both numerical and categorical data through an adaptive framework.

The major contributions, listed below, collectively emphasize the innovative hybrid data processing model, the adaptive nature of its thresholding mechanism, and the empirical validation using a Parkinson’s patient testbed, underscoring the relevance and significance of the study’s findings.

1. **Novel Hybrid Data Processing Model:** This research introduces a novel hybrid data processing model based on NRS, preserving the practical meaning of both numerical and categorical data types. Unlike conventional methods, it minimizes information loss while optimizing interpretability. The proposed distance function combines Euclidean and Levenshtein distances with weighted calculations and dynamic selection mechanisms to enhance accuracy and realism in neighborhood approximation spaces.
2. **Adaptive Thresholding Mechanism:** Another key contribution is the integration of an adaptive thresholding mechanism within the hybrid model. This feature dynamically adjusts the threshold value based on dataset characteristics, ensuring optimal performance and yielding more accurate and contextually relevant results.
3. **Empirical Validation through Parkinson’s Testbed:** This research provides a dedicated testbed for analyzing behavioral data from Parkinson’s patients, allowing rigorous evaluation of the proposed hybrid data processing model. Utilizing real-world datasets enhances the model’s practical applicability and advances knowledge in medical data analysis and diagnosis.

The subsequent structure of the paper unfolds as follows: section “Related work” delves into the related work. The proposed model is introduced in section “Adaptive neighborhood rough set model”, Section “Instrumentation” underscores the instrumentation aspect, section “Result and discussion” unfolds the presentation of results and ensuing discussions, while section “Conclusion and future work” provides the concluding remarks for the paper. A list of notations used in this study is provided in Table 1.

**Table 1:** Notations used in this study.

Notation	Detailed
SVM	Support Vector Machine
ANFIS	Adaptive Neuro-Fuzzy Inference Systems
PCA	Principal Component Analysis
NRS	Neighborhood Rough Sets
NRSs	Non-Randomized Systems
NT-MLFNRS	Noise-Tolerant Multilabel Fuzzy NRS
NRFSFN	Noise-Resistant Heuristic Multilabel Feature Selection
HIS	Hybrid Information Systems
FRS	Fitting Fuzzy Rough Set
MADM	Multiattribute Decision-Making
CVPFRSs	Covering-based Variable Precision Fuzzy Rough Sets
ANRSs	Adaptive Neighborhood Rough Sets
FSRMI	mRMR-based Feature Selection Algorithm
FNSIJE	Fuzzy Neighborhood Joint Entropy Model based on the Fuzzy Neighborhood Self-Information Measure
IvODS	Interval-Valued Ordered Decision System
*k*NN	K Nearest Neighbor
FFT	Fast Fourier Transform
UPDRS	Unified Parkinson’s Disease Rating Scale
CART	Classification and Regression Tree

## Related work

Rough set-based approaches have been utilized in various applications like bankruptcy prediction^42^, attribute/feature subset selection^43,44^, cancer prediction^45,46^, etc. In addition, recently, several innovative hybrid models have emerged, blending the realms of fuzzy logic and non-randomized systems (NRSs). One such development is presented by Yin et al.^47^, who introduce a parameterized hybrid fuzzy similarity relation. They apply this relation to the task of granulating multilabel data, subsequently extending it to the domain of multilabel learning. To construct a noise-tolerant multilabel fuzzy NRS model (NT-MLFNRS), they leverage the inclusion relationship between fuzzy neighborhood granules and fuzzy decisions. Building upon NT-MLFNRS, Yin et al. also devise a noise-resistant heuristic multilabel feature selection (NRFSFN) algorithm. To further enhance the efficiency of feature selection and address the complexities associated with handling large-scale multilabel datasets, they culminate their efforts by introducing an efficient extended version of NRFSFN known as ENFSFN.

Sang et al.^48^ explore incremental feature selection methodologies, introducing a novel conditional entropy metric tailored for dynamic ordered data robustness. Their approach introduces the concept of a fuzzy dominance neighborhood rough set (FDNRS) and defines a conditional entropy metric with robustness, leveraging the FDNRS model. This metric serves as an evaluation criterion for features, and it is integrated into a heuristic feature selection algorithm. The resulting incremental feature selection algorithm is built upon this innovative model

Wang et al.^19^ introduced the Fuzzy Rough Iterative Computational (FRIC) model, addressing challenges in hybrid information systems (HIS). Their framework includes a specialized distance function for object sets, enhancing object differentiation precision within HIS. Utilizing this function, they establish fuzzy symmetric relations among objects to formulate fuzzy rough approximations. Additionally, they introduce evaluation functions like fuzzy positive regions, dependency functions, and attribute importance functions to assess classification capabilities of attribute sets. They developed an attribute reduction algorithm tailored for hybrid data based on FRIC principles. This work contributes significantly to HIS analysis, providing a robust framework for data classification and feature selection in complex hybrid information systems.

Xu et al.^49^ introduced a novel Fitting Fuzzy Rough Set (FRS) model enriched with relative dependency complement mutual information. This model addresses challenges related to data distribution and precision enhancement of fuzzy information granules. They utilized relative distance to mitigate the influence of data distribution on fuzzy similarity relationships and introduced a fitting fuzzy neighborhood radius optimized for enhancing the precision of fuzzy information granules. Within this model, the authors conducted a comprehensive analysis of information uncertainty, introducing definitions of relative complement information entropy and formulating a multiview uncertainty measure based on relative dependency complement mutual information. This work significantly advances our understanding of managing information uncertainty within FRS models, making a valuable contribution to computational modeling and data analysis.

Jiang et al.^50^ presented an innovative approach for multiattribute decision-making (MADM) rooted in PROMETHEE II methodologies. Building upon the NRS model, they introduce two additional variants of covering-based variable precision fuzzy rough sets (CVPFRSs) by applying fuzzy logical operators, specifically type-I CVPFRSs and type-II CVPFRSs. In the context of MADM, their method entails the selection of medicines using an algorithm that leverages the identified features.

Qu et al.^51^ introduced the concept of Adaptive Neighborhood Rough Sets (ANRSs), aiming for effective integration of feature separation and linkage with classification. They utilize the mRMR-based Feature Selection Algorithm (FSRMI), demonstrating outstanding performance across various selected datasets. However, it’s worth noting that FSRMI may not consistently outperform other algorithms on all datasets.

Xu et al.^52^ introduced the Fuzzy Neighborhood Joint Entropy Model (FNSIJE) for feature selection, leveraging fuzzy neighborhood self-information measures and joint entropy to capture combined feature information. FNSIJE comprehensively analyzes the neighborhood decision system, considering noise, uncertainty, and ambiguity. To improve classification performance, the authors devised a new forward search method. Experimental results demonstrated the effectiveness of FNSIJE-KS, efficiently selecting fewer features for both low-dimensional UCI datasets and high-dimensional gene datasets while maintaining optimal classification performance. This approach advances feature selection techniques in machine learning and data analysis.

In^53^, the authors introduced a novel multi-label feature selection method utilizing fuzzy NRS to optimize classification performance in multi-label fuzzy neighborhood decision systems. By combining the NRS and FRS models a Multi-Label Fuzzy NRS model is introduced. They devised a fuzzy neighborhood approximation accuracy metric and crafted a hybrid metric integrating fuzzy neighborhood approximate accuracy with fuzzy neighborhood conditional entropy for attribute importance evaluation. Rigorous evaluation of their methods across ten diverse multi-label datasets showcased significant progress in multi-label feature selection techniques, promising enhanced classification performance in complex multi-label scenarios.

Sanget et al.^54^ introduced the Fuzzy Dominance Neighborhood Rough Set (NRS) model for Interval-Valued Ordered Decision Systems (IvODS), along with a robust conditional entropy measure to assess monotonic consistency within IvODS. They also presented two incremental feature selection algorithms. Experimental results on nine publicly available datasets showcased the robustness of their proposed metric and the effectiveness and efficiency of the incremental algorithms, particularly in dynamic IvODS updates. This research significantly advances the application of fuzzy dominance NRS models in IvODS scenarios, providing valuable insights for data analysis and decision-making processes.

Zheng et al.^55^ generalized the FRSs using axiomatic and constructive approaches. A pair of dual generalized fuzzy approximation operators is defined using arbitrary fuzzy relation in the constructive approach. Different classes of FRSs are characterized using different sets of axioms. The postulates governing fuzzy approximation operators ensure the presence of specific categories of fuzzy relations yielding identical operators. Using a generalized FRS model, Hu et al.^18^ introduced an efficient algorithm for hybrid attribute reduction based on fuzzy relations constructing a forward greedy algorithm for hybrid attribute reduction resulting in optimal classification performance with lesser selected features and higher accuracy. Considering the similarity between two objects, Wang et al.^36^ redefine fuzzy upper and lower approximations. The existing concepts of knowledge reduction are extending fuzzy environment resulting in a heuristic algorithm to learn fuzzy rules.

Gogoi et al.^56^ use rough set theory for generating decision rules from inconsistent data. The proposed scheme uses indiscernibility relation to find inconsistencies in the data generating minimized and non-redundant rules using lower and upper approximations. The proposed scheme is based on the LEM2 algorithm^57^ which performs the local covering option for generating minimum and non-redundant sets of classification rules and does not consider the global covering. The scheme is evaluated on a variety of data sets from the UCI Machine Learning Repository. All these data sets are either categorical or numerical having variable feature spaces. The proposed scheme performs consistently better for categorical data sets, as it is designed to handle inconsistencies in the data having at least one inconsistency. Results show that the proposed scheme generates minimized rule without reducing the feature space unlike other schemes, which compromise the feature space.

In^58^, the authors introduced a novel NRS model to address attribute reduction in noisy systems with heterogeneous attributes. This model extends traditional NRS by incorporating tolerance neighborhood relation and probabilistic theory, resulting in more comprehensive information granules. It evaluates the significance of heterogeneous attributes by considering neighborhood dependency and aims to maximize classification consistency within selected feature spaces. The feature space reduction algorithm employs an incremental approach, adding features while preserving maximal dependency in each round and halting when a new feature no longer increases dependency. This approach selects fewer features than other methods while achieving significantly improved classification performance, demonstrating its effectiveness in attribute reduction for noisy systems.

Zhu et al.^59^ propose a fault tolerance scheme combining kernel method, NRS, and statistical features to adaptively select sensitive features. They employ a Gaussian kernel function with NRS to map fault data to a high-dimensional space. Their feature selection algorithm utilizes the hyper-sphere radius in high-dimensional feature space as the neighborhood value, selecting features based on significance measure regardless of the classification algorithm. A wrapper deploys a classification algorithm to evaluate selected features, choosing a subset for optimal classification. Experimental results demonstrate precise determination of the neighborhood value by mapping data into a high-dimensional space using the kernel function and hyper-sphere radius. This methodology proficiently selects sensitive fault features, diagnoses fault types, and identifies fault degrees in rolling bearing datasets.

A neighborhood covering a rough set model for the fuzziness of decision systems is proposed that solves the problem of hybrid decision systems having both fuzzy and numerical attributes^60^. The fuzzy neighborhood relation measures the indiscernibility relation and approximates the universe space using information granules, which deal with fuzzy attributes directly. The experimental results evaluate the influence of neighborhood operator size on the accuracy and attribute reduction of fuzzy neighborhood rough sets. The attribute reduction increases with the increase in the threshold size. A feature will not distinguish any samples and cannot reduce attributes if the neighborhood operator exceeds a certain value.

Hou et al.^61^ applied NRS reduction techniques to cancer molecular classification, focusing on gene expression profiles. Their method introduced a novel perspective by using gene occurrence probability in selected gene subsets to indicate tumor classification efficacy. Unlike traditional methods, it integrated both Filters and Wrappers, enhancing classification performance while being computationally efficient. Additionally, they developed an ensemble classifier to improve accuracy and stability without overfitting. Experimental results showed the method achieved high prediction accuracy, identified potential cancer biomarkers, and demonstrated stability in performance.

**Table 2:** Comparison of existing schemes.

References	Comparison parameters				
	Handle hybrid data	Generalized neighborhood rough set	Attribute reduction	Classification	Accuracy
^19^	YES	NO	YES	YES	Comparable
^47^	YES	NO	YES	YES	Comparable
^48^	YES	NO	YES	YES	Comparable
^36^	YES	NO	YES	YES	Comparable
^49^	YES	NO	YES	YES	Comparable
^50^	YES	YES	YES	YES	Comparable
^51^	NO	YES	YES	YES	High
^54^	YES	YES	YES	YES	Comparable
^52^	YES	YES	YES	YES	Comparable
^53^	YES	YES	YES	YES	Comparable
^62^	NO	NO	YES	YES	Comparable
^56^	YES	NO	YES	YES	High
^58^	YES	NO	NO	YES	Comparable
^59^	NO	NO	YES	YES	Better than C4.5
^60^	YES	NO	NO	YES	Comparable
^61^	NO	NO	YES	YES	High

Table 2 gives a comparison of existing rough set-based schemes for quantitative and qualitative analysis. The comparative parameters include handling hybrid data, generalized NRS, attribute reduction, classification, and accuracy rate. Most of the existing schemes do not handle hybrid data sets without discretization resulting in information loss and a lack of practical meanings. Another parameter to evaluate the effectiveness of the existing scheme is the ability to adapt the threshold value according to the given data sets. Most of the schemes do not adapt threshold values for neighborhood approximation space resulting in variable accuracy rates for different datasets. The end-user has to adjust the value of the threshold for different datasets without understanding its impact in terms of overfitting. Selecting a large threshold value will result in more global rules resulting in poor accuracy. There needs to be a mechanism to adaptively choose the value of the threshold considering both the global and local information without compromising on the accuracy rate. The schemes are also evaluated for their ability to attribute reduction using NRS. This can greatly improve processing time and accuracy by not considering insignificant attributes. The comparative analysis shows that most of the NRS-based existing schemes perform better than many other well-known schemes in terms of accuracy. Most of these schemes have a higher accuracy rate than CART, C4.5, and *k*NN. This makes the NRS-based schemes a choice for attribute reduction and classification.

## Adaptive neighborhood rough set model

The detailed analysis of existing techniques highlights the need for a generalized NRS-based classification technique to handle both categorical and numerical data. The proposed NRS-based techniques not only handle the hybrid information granules but also dynamically select the threshold $$\delta $$ producing optimal results with a high accuracy rate. The proposed scheme considers a hybrid tuple $$HIS=\langle U_h,\ Q_h,\ V,\ f \rangle $$, where $$U_h$$ is nonempty set of hybrid records $$\{x_{h1},\ x_{h2},\ x_{h3},\ \ldots ,\ x_{hn}\}$$, $$Q_h=\left\{ q_{h1},\ q_{h2},\ \ q_{h3},\ \ldots \,\ q_{hn}\right\} $$ is the non-empty set of hybrid features. $$ V_{q_h}$$ is the domain of attribute $$q_h$$ and $$V=\ \cup _{q_h\in Q_h}V_{q_h}$$, and $$f=U_h\ x\ Q_h\rightarrow V$$ is a total function such $$f\left( x_h,q_h\right) \in V_{q_h}$$ for each $$q_h\in Q_h, x_h\in U_h$$, called information function. $$\langle U_h,\ Q_h,\ V,\ f\rangle $$ is also known as a decision table if $$Q_h=C_h\cup D$$, where $$C_h$$ is the set of hybrid condition attributes and D is the decision attribute.

A neighborhood relation *N* is calculated using this set of hybrid samples $U_h$ creating the neighborhood approximation space $\langle U_h,\ N\rangle$ which contains information granules $\left\{ \delta ({x_h}_i)\big |{x_h}_i\in U_h\right\}$ based on some distance function $\Delta$. For an arbitrary sample ${x_h}_i\in U_h$ and $B \subseteq C_h$, the neighborhood $\delta _B({x_h}_i)$ of ${x_h}_i$ in the subspace *B* is defined as $\delta _B\left( {x_h}_i\right) =\{{x_h}_j\left| {x_h}_j\right. \in U_h,\ \Delta B(x_i,x_j) \le \delta \}$. The scheme proposes a new hybrid distance function to handle both the categorical and numerical features in an approximation space.

1 $$\begin{aligned} \ \ \Delta (x_{h_{i}}, x_{h_{j}}) =\left\{ \begin{array}{ll} \left( \sum _{i=1}^{N} \left| \frac{f\left( x_{h_{1}},a_i\right) -f\left( x_{h_{2}},a_i\right) }{4\sigma }\right| ^2\right) ^\frac{1}{2},&{}\quad if \ \ numerical \\ lev(x_{h_{i}}, x_{h_{j}}) {\left\{ \begin{array}{ll} max(x_{h_{i}}, x_{h_{j}}), \ \ \ \ \ \ \ \ \ \ \ \ \ \ \ \ \ \ \ \ \ if \ \ min(x_{h_{i}}, x_{h_{j}}) = 0 \\ min {\left\{ \begin{array}{ll} {lev}_{\left( x_{h_{i}},x_{h_{j}}\right) }\left( i-1,j\right) +1 \\ {lev}_{\left( x_{h_{i}},x_{h_{j}}\right) }\left( i,\ j-1\right) +1 \ \ \ \ \ \ \ Otherwise \\ {lev}_{\left( x_{h_{i}},x_{h_{j}}\right) }\left( i-1,j-1\right) +1_{\left( x_{h_{i}}\ne x_{h_{i}} \right) } \end{array}\right. } \end{array}\right. } &{} \quad if \ \ categorical\\ \left( \sum _{i=1}^{N}{w_i\left| \frac{f\left( x_1,a_i\right) -f\left( x_2,a_i\right) }{4\sigma }\right| ^2}\right) ^\frac{1}{2}\ or\ \left( \sum _{i=1}^{N}w_i\left| lev\left( x_{h_{i}},x_{h_{j}}\right) \right. \right) ,&{}\quad weighted \ \ distance \end{array}\right. \end{aligned}$$

The proposed distance function uses Euclidean distance for numerical features and Levenshtein distance for categorical features. The distance function also takes care of the significant features calculating weighted distance for both the categorical and numerical features. The proposed algorithm dynamically selects the distance function at the run time. The use of Levenshtein distance for categorical features provides precise distance for optimal neighborhood approximation space providing better results. Existing techniques add 1 to distance if two strings do not match in calculating the distance for categorical data and add 0 otherwise. This may not result in a realistic neighborhood approximation space.

The neighborhood size depends on the threshold $\delta$. The neighborhood will contain more samples if $\delta$ is greater and results in more rules not considering the local information data. The accuracy rate of the NRS greatly depends on the selection of threshold values. The proposed scheme dynamically calculates the threshold value for any given dataset considering both local and global information. The threshold calculation formula is given below where ${min}_D$ is the minimum distance between the set of training samples and the test sample containing local information and $R_D$ is the range of distance between the set of training samples and the test sample containing the global information.

2 $$\begin{aligned} \delta \left( x_{hi}\right) ={min}_D+r.\ (R_D) \end{aligned}$$

The proposed scheme then calculates the lower and upper approximations given a neighborhood space $\langle U_h, N\rangle$ for $X \subseteq U_h$, the lower and upper approximations of *X* are defined as:

3 $$\begin{aligned} {\underline{N}}X=\left\{ x_{hi}\big |\delta \left( x_{hi}\right) \subseteq X,\ x_{hi}\in U_h\right\} \end{aligned}$$

4 $$\begin{aligned} {\overline{N}}X=\left\{ x_{hi}\big |\delta \left( x_{hi}\right) \cap X\ne 0,\ x_{hi}\in U_h\right\} \end{aligned}$$

Given a hybrid neighborhood decision table $HNDT=\langle U_h,\ C_h\cup \ D, V, f\rangle$, $\{ X_{h1},X_{h2},\ \ldots ,\ X_{hN} \}$ are the sample hybrid subjects with decision 1 to *N*, $\delta _B\left( x_{hi}\right)$ is the information granules generated by attributes $B \subseteq C_h$, then the lower and upper approximation is defined as:

5 $$\begin{aligned} \underline{N_B}X= & {} \bigcup _{i=1}^{N}{\underline{N_B}X_{hi}} \end{aligned}$$

6 $$\begin{aligned} \overline{N_B}X= & {} \bigcup _{i=1}^{N}{\overline{N_B}X_{hi}} \end{aligned}$$

and the boundary region of *D* is defined as:

7 $$\begin{aligned} BN\left( D\right) =\overline{N_B}D-\underline{N_B}D \end{aligned}$$

The lower and upper approximation spaces are the set of rules, which are used to classify a test sample. A test sample forms its neighborhood using a lower approximation having all the rules with a distance less than a dynamically calculated threshold value. The majority voting is used in the neighborhood of a test sample to decide the class of a test sample. K-fold cross-validation is used to measure the accuracy of the proposed scheme where the value k is 10. The algorithm 1 of the proposed scheme has a time complexity of $O(nm^{2})$ where *n* is the number of clients and *m* is the size of the categorial data.
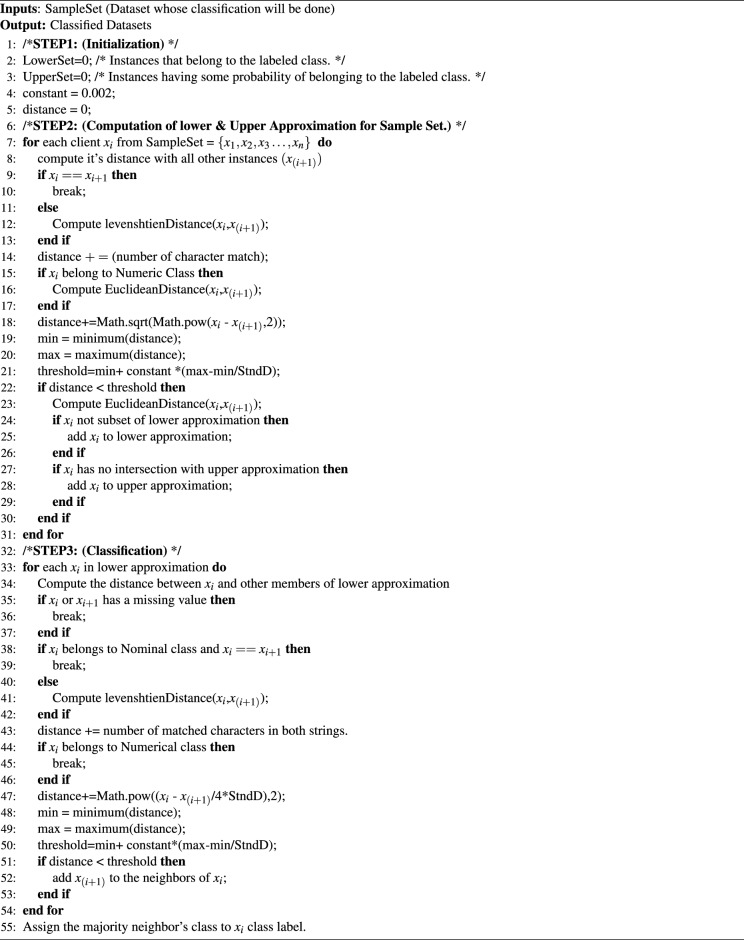


## Instrumentation

The proposed generalized rough set model has been rigorously assessed through the development of a testbed designed for the classification of Parkinson’s patients. It has also been subjected to testing using various standard datasets sourced from the University of California at Irvine machine learning data repository^63^. This research underscores the increasing significance of biomedical engineering in healthcare, particularly in light of the growing prevalence of Parkinson’s disease, which ranks as the second most common neurodegenerative condition, impacting over 1% of the population aged 65 and above^64^. The disease manifests through distinct motor symptoms like resting tremors, bradykinesia (slowness of movement), rigidity, and poor balance, with medication-related side effects such as wearing off and dyskinesias^65^.

In this study, to address the need for a reliable quantitative method for assessing motor complications in Parkinson’s patients, the data collection process involves utilizing a home-monitoring system equipped with wireless wearable sensors. These sensors were specifically deployed to closely monitor Parkinson’s patients with severe tremors in real time. It’s important to note that all patients involved in the study were clinically diagnosed with Parkinson’s disease. Additionally, before data collection, proper consent was obtained from each participant, and the study protocol was approved by the ethical committee of our university. The data collected from these sensors is then analyzed, yielding reliable quantitative information that can significantly aid clinical decision-making within both routine patient care and clinical trials of innovative treatments.

**Figure 1:**
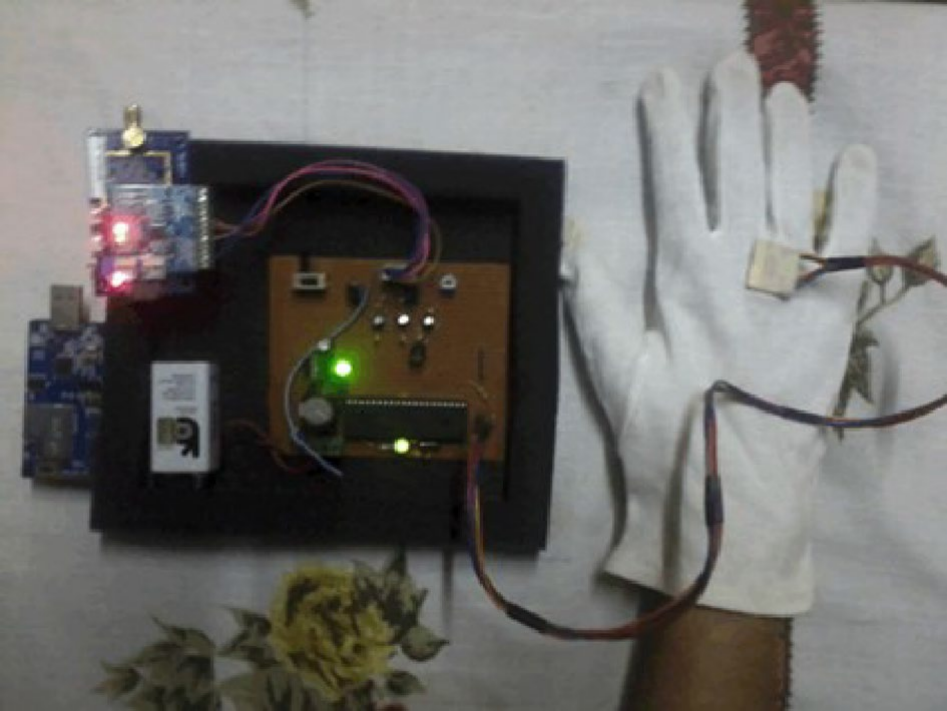
Testbed for Parkinson’s patients.

**Figure 2:**
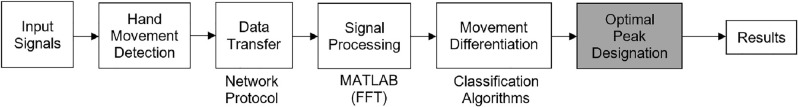
Experimental flowchart.

Figure 1 illustrates a real-time Testbed designed for monitoring Parkinson’s patients. This system utilizes a tri-axial accelerometer to capture three signals, one for each axis $(x,\ y,\ and\ z)$, resulting in a total of 18 channels of data. The sensors employed in this setup employ ZigBee (IEEE 802.15.4 infrastructure) protocol to transmit data to a computer at a sampling rate of 62.5 Hz. To ensure synchronization of the transmitted signals, a transition protocol is applied. These data packets are received through the Serial Forwarder using the TinyOS platform (http://www.tinyos.net). The recorded acceleration data is represented as digital signals and can be visualized on an oscilloscope. The frequency domain data is obtained by applying the Fast Fourier Transform (FFT) to the signal, resulting in an ARFF file format that is then employed for classification purposes. The experimental flowchart is shown in Fig. 2.

The real-time testbed includes various components to capture data using the Unified Parkinson’s Disease Rating Scale (UPDRS). TelosB MTM-CM5000-MSP and MTM-CM3000-MSP sensors are used to send and receive radio signals from the sensor to the PC. These sensors are based on an open-source TelosB/Tmote Sky platform, designed and developed by the University of California, Berkeley.

TelosB sensor uses the IEEE 802.15.4 wireless structure and the embedded sensors can measure temperature, relative humidity, and light. In CM3000, the USB connector is replaced with an ERNI connector that is compatible with interface modules. Also, the Hirose 51-pin connector makes this more versatile as it can be attachable to any sensor board family, and the coverage area is increased using SMA design by a 5dBi external antenna^66^. These components can be used for a variety of applications such as low-power Wireless Sensor Networks (WSN) platforms, network monitoring, and environment monitoring systems.

MTS-EX1000 sensor board is used for the amplification of the voltage/current value from the accelerometer. The EX1000 is an attachable board that supports the CMXXXX series of wireless sensors network Motes (Hirose 51-pin connector). The basic functionality of EX1000 is to connect the external sensors with CMXX00 communication modules to enhance the mote’s I/O capability and support different kinds of sensors based on the sensor type and its output signal. ADXL-345 Tri-accelerometer sensor is used to calculate body motion along x, y, and z-axis relative to gravity. It is a small, thin, low-power, 3-axis accelerometer that calculates high resolution (13-bit) measurements at up to ±16g. Its digital output, in 16-bit twos complement format, is accessible through either an SPI (3- or 4-wire) or I2C digital interface. A customized main circuit board is used having a programmed IC, registers, and transistors. Its basic functionality is to convert the digital data, accessed through the ADXL-345 sensor, into analog form and send it to MTS1000.

## Result and discussion

The proposed generalized and ANRS is evaluated against different data sets taken from the machine learning data repository, at the University of California at Irvine. In addition to these common data sets, a real-time Testbed for Parkinson’s patients is also used to evaluate the proposed scheme. The hybrid data of 500 people was collected using the Testbed for Parkinson’s patients including 10 Parkinson’s patients, 20 people have abnormal and uncontrolled hand movements, and the rest of the samples were taken approximating the hand movements of Parkinson’s patients. The objective of this evaluation is to compare the accuracy rate of the proposed scheme with CART, *k*NN, and SVM having both simple and complex datasets containing numerical and hybrid features respectively. The results also demonstrate the selection of radius r for dynamically calculating the threshold value.

Table 3 provides the details of the datasets used for the evaluation of the proposed scheme including the training and test ratio used for evaluation in addition to data type, total number of instances, total feature, a feature considered for evaluation, and number of classes. The hybrid datasets are also selected to evaluate to performance of the proposed scheme against the hybrid feature space without discretization preventing information loss.

**Table 3:** Summary of datasets used for evaluation.

Name	Type	Instances	Train:test ratios	No. of features	Classes
Bupa^67^	Real	345	200:145	6	2
Sonar^68^	Real	208	100:108	60	2
Mammographic Mass^69^	Real	961	516:445	6	2
Haberman’s Survival^70^	Real	306	200:106	3	2
Credit-g^71^	Real	1000	640:360	20	2
Oil Spill^72^	Real	937	600:337	48	2
Lymmography^73^	Hybrid	148	70:30	18	2
Splice^74^	Real	3190	2233:957	61	2
Optdigits^75^	Real	5620	3823:1797	64	2
Pendigits^76^	Real	9868	6908:2960	16	2
Pageblocks^77^	Real	5473	3831:1642	10	2
Statlog^78^	Real	6435	4505:1930	36	2
Magic04^79^	Real	19020	13314:5706	10	2
Parkinson’s	Hybrid	500	350:150	10	2

The accuracy of the NRS is greatly dependent on the threshold value. Most of the existing techniques do not dynamically adapt the threshold $\delta$ value for different hybrid datasets. This results in the variant of NRS suitable for specific datasets with different threshold values. A specific threshold value may produce better results for one dataset and poor results for others requiring a more generic threshold value catering to different datasets with optimal results. The proposed scheme introduces an adaptable threshold calculation mechanism to achieve optimal results regardless of the datasets under evaluation. The radius value plays a pivotal role in forming a neighborhood, as the threshold values consider both the local and global information of the NRS to calculate neighborhood approximation space. Table 4 shows the accuracy rate having different values of the radius of the NRS. The proposed threshold mechanism provides better results for all datasets if the value of the radius is 0.002. Results also show that assigning no weight to the radius produces poor results, as it will then only consider the local information for the approximation space. Selecting other weights for radius may produce better results for one dataset but not for all datasets.

**Table 4:** Accuracy rate with different values of “R”.

Dataset	Fixed weight (%)	No weight (%)	Adaptive weight (%) (with $R=0.002$)
Bupa^67^	87	0	98
Credit-g^71^	81	0	100
Oil-spill^72^	98	0	98
Mammographic Mass^69^	83	0	100
Sonar^68^	89	0	82
Haberman’s survival^70^	73	73	73
Lymmgraphy^73^	76	0	100

Table 5 presents the comparative analysis of the proposed scheme with *k*NN, Naive Bayes, and C45. The results show that the proposed scheme performs well against other well-known techniques for both the categorical and numerical features space. Naive Bayes and C45 also result in information loss, as these techniques cannot process the hybrid data. So the proposed scheme handles the hybrid data without compromising on the information completeness producing acceptable results. K-fold cross-validation is used to measure the accuracy of the proposed scheme. Each dataset is divided into 10 subsets to use one of the K subsets as the test set and the other K-1 subsets as training sets. Then the average accuracy of all K trials is computed with the advantage of having results regardless of the dataset division.

**Table 5:** Comparative analysis of the proposed scheme.

Name	Type	*k*NN (%)	Naive Bayes (%)	C45 (%)	Proposed scheme (%)
Bupa^67^	Real	60	52	66	98
Sonar^68^	Real	76	69	75	82
Mammographic mass^69^	Real	82	82	81	100
Haberman’s survival^70^	Real	72	74	71	73
Credit-g^71^	Real	72	75	71	100
Oil spill^72^	Real	96	88	95	98
Lymmography^73^	Hybrid	82	85	84	100
Splice^74^	Real	97	99	100	100
Optdigits^75^	Real	100	97	99	98
Pendigits^76^	Real	98	98	99	100
Pageblocks^77^	Real	97	94	99	100
Statlog^78^	Real	98	90	99	100
Magic04^79^	Real	100	100	100	100
Parkinson’s	Hybrid	85	87	86	95

## Conclusion and future work

This work evaluates the existing NRS-based scheme for handling hybrid data sets i.e. numerical and categorical features. The comparative analysis of existing NRS-based schemes shows that there is a need for a generic NRS-based approach to adapt the threshold selection forming neighborhood approximation space. A generalized and ANRS-based scheme is proposed to handle both the categorical and numerical features avoiding information loss and lack of practical meanings. The proposed scheme uses a Euclidean and Levenshtein distance to calculate the upper and lower approximation of NRS for numerical and categorical features respectively. Euclidean and Levenshtein distances have been modified to handle the impact of outliers in calculating the approximation spaces. The proposed scheme defines an adaptive threshold mechanism for calculating neighborhood approximation space regardless of the data set under consideration. A Testbed is developed for real-time behavioral analysis of Parkinson’s patients evaluating the effectiveness of the proposed scheme. The evaluation results show that the proposed scheme provides better accuracy than *k*NN, C4.5, and Naive Bayes for both the categorical and numerical feature space achieving 95% accuracy on the Parkinson’s dataset. The proposed scheme will be evaluated against the hybrid data set having more than two classes in future work. Additionally, in future work, we aim to explore the following areas; (i) conduct longitudinal studies to track the progression of Parkinson’s disease over time, allowing for a deeper understanding of how behavioral patterns evolve and how interventions may impact disease trajectory, (ii) explore the integration of additional data sources, such as genetic data, imaging studies, and environmental factors, to provide a more comprehensive understanding of Parkinson’s disease etiology and progression, (iii) validate our findings in larger and more diverse patient populations and investigate the feasibility of implementing our proposed approach in clinical settings to support healthcare providers in decision-making processes, (iv) investigate novel biomarkers or physiological signals that may provide additional insights into Parkinson’s disease progression and motor complications, potentially leading to the development of new diagnostic and monitoring tools, and (v) conduct patient-centered outcomes research to better understand the impact of Parkinson’s disease on patients’ quality of life, functional abilities, and overall well-being, with a focus on developing personalized treatment approaches.

## Data Availability

The datasets used in this study are publicly available at the following links: Bupa^67^: 10.24432/C54G67, Sonar^68^: 10.24432/C5T01Q, Mammographic Mass^69^: 10.24432/C53K6Z, Haberman’s Survival^70^: 10.24432/C5XK51, Credit-g^71^: 10.24432/C5NC77, Lymmography^73^: 10.24432/C54598, Splice^74^: 10.24432/C5M888, Optdigits^75^: 10.24432/C50P49, Pendigits^76^: 10.1137/1.9781611972825.9, Pageblocks^77^: 10.24432/C5J590, Statlog^78^: 10.24432/C55887, Magic04^79^: 10.1609/aaai.v29i1.9277.
